# Heparan Sulfate as a Therapeutic Target in Tauopathies: Insights From Zebrafish

**DOI:** 10.3389/fcell.2018.00163

**Published:** 2018-12-20

**Authors:** Seyedeh Maryam Alavi Naini, Nadia Soussi-Yanicostas

**Affiliations:** ^1^Department of Neuroscience, Institut de Biologie Paris Seine (IBPS), INSERM, CNRS, Sorbonne Université, Paris, France; ^2^PROTECT, INSERM U1141, Université Paris Diderot, Sorbonne Paris Cité, Paris, France

**Keywords:** heparan sulfate, glycosaminoglycans, tauopathy, tau, Alzheimer’s disease, zebrafish, drug discovery

## Abstract

Microtubule-associated protein tau (MAPT) hyperphosphorylation and aggregation, are two hallmarks of a family of neurodegenerative disorders collectively referred to as tauopathies. In many tauopathies, including Alzheimer’s disease (AD), progressive supranuclear palsy (PSP) and Pick’s disease, tau aggregates are found associated with highly sulfated polysaccharides known as heparan sulfates (HSs). In AD, amyloid beta (Aβ) peptide aggregates associated with HS are also characteristic of disease. Heparin, an HS analog, promotes misfolding, hyperphosphorylation and aggregation of tau protein *in vitro*. HS also provides cell surface receptors for attachment and uptake of tau seeds, enabling their propagation. These findings point to HS-tau interactions as potential therapeutic targets in tauopathies. The zebrafish genome contains genes paralogous to MAPT, genes orthologous to HS biosynthetic and chain modifier enzymes, and other genes implicated in AD. The nervous system in the zebrafish bears anatomical and chemical similarities to that in humans. These homologies, together with numerous technical advantages, make zebrafish a valuable model for investigating basic mechanisms in tauopathies and identifying therapeutic targets. Here, we comprehensively review current knowledge on the role of HSs in tau pathology and HS-targeting therapeutic approaches. We also discuss novel insights from zebrafish suggesting a role for HS 3-*O*-sulfated motifs in tau pathology and establishing HS antagonists as potential preventive agents or therapies for tauopathies.

## Heparan Sulfate Proteoglycans

### Structure and Biosynthesis

Glycosaminoglycans (GAGs), formerly called mucopolysaccharides, are a major class of anionic polysaccharides, consisting of unbranched and often long polysaccharide chains made of disaccharide units. Most GAGs are bound to core proteins, forming proteoglycans (PGs), which are important components of extracellular matrices. All extracellular matrices contain PGs, but these glycoproteins are also found in membrane-bound secretory granules and in cell nuclei. PGs are evolutionarily ancient and are found in all Bilateria species (organisms with left-right symmetry). So far, five structurally different GAG species have been described: heparan sulfate (HS), chondroitin sulfate (CS), dermatan sulfate (DS), keratan sulfate (KS) and hyaluronan or hyaluronic acid (HA). While heparin, a highly sulfated HS, is mainly produced by connective tissue-type mast cells and bipotential glial progenitor cells, HS is synthesized by all cell types (Couchman and Pataki, 2012; Lindahl et al., 2017). In the zebrafish (*Danio rerio*), HS is synthetized in developing embryos, larvae and adults (Zhang et al., 2009).

Glycosaminoglycans synthesis starts in the endoplasmic reticulum (ER), with the formation of a tetrasaccharide linker (Xyl-Gal-Gal-GlcA) bound to the core protein via a serine residue. During linker formation, xylose (Xyl) is first attached by a xylosyltransferase (XT); galactosyl-transferases I and II (GT I and II) then transfer two galactoses (Gal) from UDP-Gal to xylose, and finally, glucoronic acid (GlcA) is transferred from UDP-GlcA by glucuronosyl-transferase I (GlcAT). After the formation of the linker, GAG biosynthesis takes different routes for CS/DS or heparin/HS synthesis. Synthesis of CS/DS chains first involves the transfer of *N*-acetylgalactosamine (GalNAc) by GalNAc transferase to the terminal GlcA, while that of heparin/HS chains depends on the transfer of *N*-acetylglucosamine (GlcNAc) by GlcNAc transferase. The GAG chains are then elongated by sequential addition of repeated disaccharide units composed of an amino sugar, [*N*-acetylgalactosamine (GalNAc) or *N*-acetylgalactosamine (GalNAc)], and a uronic acid [glucoronic acid (GlcA) or iduronic acid (IdoA)]. In HS and heparin, the repeated disaccharide unit comprises *N*-acetylglucosamine and glucoronic acid residues. EXT enzymes mediate HS/heparin chain elongation (Kreuger and Kjellén, 2012).

After their synthesis, GAG chains are modified in the Golgi apparatus by different enzymes. All HS chain modifier enzymes have been identified in zebrafish (Bink et al., 2003; Chen et al., 2005; Ghiselli and Farber, 2005; Cadwallader and Yost, 2006a,b, 2007; Filipek-Górniok et al., 2015). Major modifications on the HS chains are sulfations, which are mediated by two classes of sulfotransferases in a hierarchical manner. The expression patterns of these enzymes are characterized in zebrafish through early developmental stages (0–48 hpf). First, the *N*-deacetylase/*N*-sulfotransferases (NDSTs) replace the acetyl group in GlcNAc by a sulfate group. Five NDST genes (*ndst1a, ndst1b, ndst2a, ndst2b, and ndst3*) have been identified in zebrafish. All isoforms are expressed in various brain regions during early zebrafish development, while a distinct isoform (*ndst3*) is expressed in the spinal cord (Filipek-Górniok et al., 2015). HS chains can be further modified through epimerization of GlcA into iduronic acid (IdoA) by C-5 epimerase. The zebrafish C-5 epimerases Glce-A and Glce-B are expressed in brain through early embryonic development (Cadwallader and Yost, 2007).

The second class of sulfotransferases comprises the HS *O*-sulfotransferases (OSTs) 2-OST, 6-OST, and 3-OST. Zebrafish 2-OST or HS2ST is expressed in all brain regions in early developmental stages. Of the four zebrafish 6-OSTs or HS6STs (6-OST-1a, 6-OST-1b, 6-OST-2, and 6-OST-3) all are expressed in the brain in early development, while only 6-OST-1a and 6-OST-1b isoforms are expressed in the spinal cord (Cadwallader and Yost, 2006b). Comparison of HS structure between adult zebrafish and porcine intestine revealed similar global structures. Differences were documented as higher 2-*O*-sulfated iduronic acid (IdoA2S) content and lower levels of GlcA (Zhang et al., 2009).

It has been suggested that the evolutionary origin of the Hs3sts goes back to a common ancestor of bilaterians and cnidarians in the early eumetazoan evolution. *Trichoplax*, a placozoan identified as a sister clade to bilaterians and cnidarians, has no Hs3st homologs, but expresses all the other HS sulfotransferases. Invertebrates express fewer Hs3st isozymes than vertebrates. *Hydra* expresses one Hs3st, whereas *Nematostella* (sea anemone), belonging to the Cnidarian phylum, expresses two Hs3sts. The Hs3st enzymes in *Hydra* and *Nematostella* share near 50% amino acid sequence identity with human Hs3sts. *Strongylocentrotus* (sea urchin) and *Planaria* (flatworm) express a single Hs3st, whereas *Drosophila* (fruit fly) and *Caenorhabditis* (nematode) have two Hs3st. Vertebrates express a greater number of Hs3sts. *Homo* and other mammals express 7 Hs3sts, while *Danio* (zebrafish) expresses 8 (Thacker et al., 2014). 3-*O*-Sulfated disaccharides and tetrasaccharides have been identified in HS from zebrafish embryos, which were diminished by morpholinos directed against specific *3-ost* genes. During early cleavage stages in the zebrafish, 3-OST enzymes are encoded by maternally deposited mRNAs, which are evenly distributed throughout the embryos. By contrast, during later stages, and particularly in the developing brain, the zebrafish Hs3st genes display a complex combinatorial expression profile with each gene showing a unique and cell-type specific expression pattern. The *3ost1* transcripts are ubiquitously accumulated to high levels during early cleavage stages prior to becoming restricted to head and anterior somites by 24 hpf. In 48 hpf embryos, *3ost1* expression is restricted to head, gut and pectoral fin. Expression of the *3ost2* gene is detected from 24 hpf onward in the brain and otic vesicle. At 48 hpf, *3ost2* is expressed in all major brain areas, as well as in the olfactory region. Zebrafish *3ost3X* and *-3Z* are the orthologs of the mammalian *3-OST-3a* and *-3b* genes, respectively. *3ost3X* transcripts are evenly distributed during early cleavage stages and are restricted to hindbrain and spinal cord from 36 hpf onward. By contrast, while expression of the *3ost3Z* gene is not detected during early cleavage stages, by 48 hpf it is transcribed in telencephalon, tectal regions, hindbrain, and spinal cord. *3ost4* RNAs are maternally expressed and ubiquitously accumulated in early cleavage stage embryos, before being specifically transcribed in midbrain, hindbrain, olfactory epithelium and otic vesicle by 48 hpf onward. Similarly, ubiquitous accumulation of maternal *3ost5* transcripts is detected in early cleavage and embryonic stages, while from mid-somitogenesis onward, these RNAs are specifically transcribed in forebrain, midbrain and spinal cord neurons. Distribution of *3ost6* transcripts is detected during early cleavage stages, while from 36 hpf onward these RNAs are expressed in the hindbrain. Finally, the *3ost7* gene is expressed during early cleavage stages and becomes restricted to brain neurons by 48 hpf (Cadwallader and Yost, 2006a). The zebrafish *3ost2* and *3ost3* generate receptors for herpes simplex virus 1 (HSV1) entry, highlighting the functionality and similarity of zebrafish neuronal 3-*O*-sulfated motifs to those found in humans (Antoine et al., 2014).

Heparan sulfate PGs (HSPGs) comprise one or more HS-sulfated GAG chains covalently linked to a core protein. 17 distinct types of HSPGs have been identified, which are classified into three groups based on their locations: (i) syndecans and glypicans (membrane HSPGs), (ii) agrin, perlecan, and type-XVIII collagen (secreted extracellular matrix HSPGs), and (iii) serglycin (secretory vesicle HSPG) (Lindahl et al., 2017). Agrin also occurs as a transmembrane proteoglycan resulting from alternative splicing (Burgess et al., 2000; Neumann et al., 2001). The structure and function of zebrafish syndecan-4, required for neural crest migration, closely resembles that found in higher vertebrates (Whiteford et al., 2008). Using brains of BACE-1 deficient zebrafish, glypican-1 is identified as a substrate for the enzyme. BACE-1 is implicated in pathogenesis of AD by contributing to generation of Aβ peptide (Hogl et al., 2013).

Heparan sulfate chains of HSPGs can also be modified post-assembly by several enzymes. In particular, the shortening and degradation of HS chains involve heparanase (HPSE), and the removal of sulfate motifs by SULFs (a family of plasma membrane endosulfatases) entails finer modifications. A single gene orthologous to human heparanase is found in zebrafish (Wei and Liu, 2014). Orthologs to mammalian SULFs have been identified in zebrafish; they consist of three genes, all expressed in the central nervous system (CNS) (Gorsi et al., 2010). PG degradation is mediated by their internalization, which induces endolytic cleavage of the core protein by proteases and degradation of heparan sulfate chains by heparanase and endo-β-glucuronidase. Complete degradation of the remaining small HS chains takes place in lysosomes and involves a series of exoglycosidases and sulfatases (Lamanna et al., 2008; Lindahl et al., 2017). The ortholog of the human iduronate-2-sulfatase in zebrafish, whose defects are responsible for mucopolysaccharidosis type II (MPS II), has been identified (Moro et al., 2010). The identification of HSs in zebrafish, together with an array of orthologs for HS biosynthetic and chain modifier enzymes in the nervous system, makes zebrafish a model organism well-suited for the study of HS contribution to tauopathies.

### HS-Protein Interactions

Heparan sulfates can bind non-covalently to a variety of proteins. An array of heparin-binding proteins have been identified, many of which interact with HSs under physiological conditions and modulate their biological activities (Bishop et al., 2007; Ori, 2008; Xu and Esko, 2014). Protein-HS interactions promote protein presentation, protection and stabilization, as well as conformational changes and oligomerization (Thacker et al., 2014). The conformation of GAG chains and the negative charges provided by the sulfate groups and uronic acid epimers constitute the binding site for positively charged amino acids of heparin-binding proteins (Lindahl and Li, 2009; Sarrazin et al., 2011). The ligand-binding sites of HSPGs rely on the orientation of carboxyl groups and organization of sulfate groups (Sarrazin et al., 2011). In particular, the sulfation pattern of HSPGs, which is more diverse than other GAG chain modifications, plays an essential role in the specificity of the binding sites of HSPGs to heparin-binding proteins (Aviezer et al., 1994; Sanderson et al., 1994; Herndon et al., 1999). Moreover, it has been shown that different patterns of sulfation in HSPGs with similar core proteins lend them different binding specificities (Kato et al., 1994; Sanderson et al., 1994). Hence the concerted action of enzymes confers highly variable patterns of sulfation to HS chains, thus driving their complexity and micro-heterogeneity (Shriver et al., 2012; Thacker et al., 2014). Importantly, data suggest that GAG chain sulfation does not go to completion on sulfated polysaccharide chains, yielding alternate highly sulfated domains (NS domains) and regions showing low or no sulfation (NA domains) (Sarrazin et al., 2011).

The sulfation pattern of most HSPGs changes during development, thus modifying their binding specificities (Nurcombe et al., 1993; Brickman et al., 1998; Yabe et al., 2005), a process playing a critical role in nervous system development (Irie et al., 2002; Bülow and Hobert, 2004). HS modifications dynamically evolve during zebrafish development (Cadwallader and Yost, 2006a,b, 2007; Zhang et al., 2009). Studies in zebrafish have demonstrated a critical requirement for HS in axon pathfinding during development (Lee et al., 2004; Kim et al., 2007; Kastenhuber et al., 2009; Wang et al., 2012; Poulain and Chien, 2013). Chemical disruption of HS and CS biosynthesis in early zebrafish development leads to brain disorganization (Beahm et al., 2014). However, the specific binding motifs for many heparin-binding proteins remain to be defined (Thacker et al., 2014). Full knowledge of HS-protein interactions has important therapeutic implications. Surfen, a non-specific heparan sulfate antagonist interferes with numerous biological processes, showing potential as a therapeutic agent. The properties of surfen ranges from anti-inflammatory (Warford et al., 2018) and immuomodulatory (Warford et al., 2014) to suppression of stem cell differentiation (Huang et al., 2018), inhibition of HIV type 1 infection (Roan et al., 2010) and rescue of tauopathy in a zebrafish model (Alavi Naini et al., 2018). However, non-specificity limits the beneficial effects of surfen. Surfen is reported to decrease neuroinflammation through HSPGs while increasing both the beneficial and harmful CS proteoglycans involved in remyelination (Warford et al., 2018). Thus complete knowledge of the PG interactome promotes development of specific therapies targeting harmful intractions and/or enhancing beneficial ones.

## Tau and Tauopathies

Tauopathies are neurodegenerative disorders characterized by microtubule-associated protein tau (MAPT) hyperphosphorylation and aggregation into paired helical filaments (PHFs) or straight filaments (SFs), forming neurofibrillary tangles (NFTs) in brain. Unlike amyloid-beta (Aβ) aggregates, which are specifically detected in AD patients, tau tangles are found in many neurodegenerative disorders such as corticobasal degeneration (CBD), progressive supranuclear palsy (PSP), Pick’s disease, dementia pugilistica, frontotemporal dementia with parkinsonism linked to chromosome 17 (FTDP-17), and many others including AD (Alavi Naini and Soussi-Yanicostas, 2015). *MAPT* gene mutations are identified in a number of tauopathies. The mutations alter the amino acid sequence of tau, disrupt splicing or both (Alavi Naini and Soussi-Yanicostas, 2015). AD and other dementias are currently major challenges for health care systems, and major public health priorities worldwide, for which there is an unmet need for disease-modifying treatments (Frankish and Horton, 2017).

The tau protein is identified as a microtubule-associated protein (MAP) with a wide range of potential functions. In the adult human CNS six different tau isoforms are expressed (Goedert et al., 1989b). They are generated *via* alternative splicing of a single *MAPT* gene located on chromosome 17q21.31 and comprising 16 exons (Neve et al., 1986). Tau isoforms range in length from 352 to 441 amino acids, with a molecular weight of 45–65 kDa. The exons E1, E4, E5, E7, E9, E11, E12 and E13, are included in all tau isoforms. Tau isoforms are differentiated by three (3R) or four (4R) carboxy-terminal tandem repeats of 31 amino acids, which are designated as microtubule-binding domains (MBDs). These repeated domains, which show strong sequence conservation, are encoded by exons E9, E10, E11, and E12, the exclusion or inclusion of exon 10 generates the 3R or 4R tau isoforms, respectively (Goedert et al., 1989a,b; Andreadis et al., 1992).

The main functions recognized for tau are microtubule stabilization and polymerization. Microtubules are part of the eukaryotic cytoskeletal framework and are primarily composed of α- and β-tubulin, forming tubular polymers. Microtubules are essential for cytoskeletal maintenance and intracellular transport (Nogales, 2001). Tau mutations alter their affinity for interaction and binding with microtubules (Alavi Naini and Soussi-Yanicostas, 2015). Tau is able to bind to the outside and probably to the inside of microtubules, while the N- and C-terminal regions project outward (Kar et al., 2003; Santarella et al., 2004). The tandem repeat sequences in the (MBD) provide a net positive charge that interacts directly with the negatively charged residues of tubulin (Kar et al., 2003; Jho et al., 2010). The 4R-tau isoforms have higher affinity for binding to microtubules than 3R-tau and are more efficient at promoting microtubule assembly, likely due to the presence of the inter-repeat sequences between the first and second microtubule-binding repeats, which possess over twice the binding affinity of any individual microtubule-binding repeat (Goedert and Jakes, 1990; Goode and Feinstein, 1994).

Tau alterations as biomarkers for dementia have mainly been studied in AD. The amount of total tau (T-tau) in CSF correlates with the intensity of neurodegeneration (Gao et al., 2018) and hyperphosphorylated tau (P-tau) levels in CSF correlate with hippocampal atrophy in prodromal AD (De Leon et al., 2006; Fagan and Holtzman, 2010). Interestingly, Aβ peptide accumulation does not correlate with neurodegeneration in prodromal AD (Iaccarino et al., 2018). Moreover the mean content of abnormally phosphorylated tau from several brain regions in individual AD patients closely correlates with disease severity (Holzer et al., 1994). These observations along with tau mutations responsible for a number of tauopathies, highlight the importance of tau alterations as therapeutic targets in tauopathies.

## Role of Heparan Sulfate (HS) in Tauopathies

In 1855 Virchow reported the presence of polysaccharides in amyloid deposits in brain (Virchow, 1855). In 1942 Hass characterized the polysaccharides in amyloid lesions as sulfated amino-sugar based polysaccharides, later called heparin-like glycosaminoglycans (GAGs), and then heparan sulfate proteoglycans (HSPGs) (Hass, 1942). Tauopathies are amyloid disorders (Iadanza et al., 2018). Amyloid disorders are a diverse group of protein-misfolding disorders (PMDs) characterized by disease-specific protein aggregates containing heparan sulfate proteoglycans (HSPGs) in most cases (Ancsin, 2003; Nishitsuji and Uchimura, 2017).

The association of GAGs with protein aggregates in Alzheimer’s disease (AD) and other tauopathies was described decades ago in the literature (Kato et al., 1991; Su et al., 1992; DeWitt et al., 1993, 1994). Further investigations demonstrated that sulfated GAGs played a critical role in tau hyperphosphorylation and aggregation, but also amyloid precursor protein (APP) cleavage and the resulting Aβ peptide fibrillization (Fraser et al., 1992; Brunden et al., 1993; Goedert et al., 1996; Hasegawa et al., 1997; Scholefield et al., 2003; Beckman et al., 2006). However, the nature of the sulfated GAGs involved in these processes remained unclear. In particular, GAG-tau interactions have received little attention as potential therapeutic targets.

Heparan sulfate proteoglycans are associated with both amyloid-beta (Aβ) plaques and neurofibrillary tangles (NFTs) in Alzheimer’s disease (AD) (Snow et al., 1990; Kato et al., 1991; Perlmutter et al., 1991; Perry et al., 1991; Su et al., 1992), the most frequent form of tauopathy (Hernández et al., 2018). While the principal HSPGs accumulating in the AD-associated aggregates are membrane-associated HSPGs, the presence of perlecan (a non-membrane-associated HSPG) in Aβ deposits is debated (Van Gool et al., 1993; Snow et al., 1994; Van Horssen et al., 2002). Specifically, the initially reported association of perlecan with Aβ plaques and neurofibrillary tangles in AD was later challenged because of the cross-reactivity of the antibodies used (Verbeek et al., 1999). Perlecan is increased in AD brains (Liu et al., 2016). However, a study of the perlecan mRNA levels in the hippocampus of AD patients and age-matched controls has shown similar expression levels (Maresh et al., 1996), and mice overexpressing the perlecan core protein did not develop plaques or tangles (Hart et al., 2001). Agrin is strongly expressed in the hippocampus and amygdala (Bowe and Fallon, 1995; Donahue et al., 1999), two areas strongly affected in AD (Braak and Braak, 1991). Agrin is the major HSPG associated with both Aβ plaques and tau-containing NFTs in AD (Verbeek et al., 1999; Cole and Liu, 2006). Last, heparanase (HPSE) is overexpressed in brain areas showing degeneration in AD as measured by RT-PCR (García et al., 2017), suggested be a protective mechanim. Thus membrane-associated HSPGs provide a link between the two major hallmarks of AD, Aβ plaques and NFTs. This suggests that the membrane-associated HSPGs might be involved in processes upstream of AD lesions.

In many neuropathies, abnormal HSPG accumulations are often observed at early stages of the disease. HSPGs accumulate in neuronal cell bodies in Down syndrome patients years before the first clinical symptoms of dementia occur. HSPGs are also associated with Aβ deposits in the early stages of AD. Specifically, HSPGs are associated with both immature diffuse Aβ plaques (Snow et al., 1990, 1994; Su et al., 1992; Cotman et al., 2000) and hyperphosphorylated tau aggregates, during the earliest stages of neurofibrillary pathology (Spillantini et al., 1999). Interestingly, in this situation HSPG staining is more intense than that of tau, suggesting that HSPGs play a role in initiation of disease.

In 1996 *in vitro* biochemical investigations demonstrated that interactions between heparin and tau protein led to the assembly of tau into NFT-like filaments (Goedert et al., 1996). Moreover, sulfated motifs on the GAG chain are critical for tau aggregation, as the non-sulfated hyaluronic acid does not enhance tau polymerization (Arrasate et al., 1997). Aside from structural changes, heparin was also shown to markedly stimulate the phosphorylation of tau by different protein kinases, leading to tau hyperphosphorylation, a key feature of tauopathies. In particular, tau phosphorylation by cdc28, cAMP-dependent protein kinase, GSK3b and several stress-activated protein kinases (SAP kinases) are markedly stimulated *in vitro* by heparin (Mawal-Dewan et al., 1992; Brandt et al., 1994; Yang et al., 1994; Goedert et al., 1997; Hasegawa et al., 1997). Heparin also prevents tau binding to taxol-stabilized microtubules and promotes disassembly of microtubules formed by tau and tubulin, suggesting that highly sulfated GAGs compete with microtubules for tau binding (Goedert et al., 1996). However, further research showed that the effect of heparin on tau was mimicked by several GAGs and depended on their degree of sulfation: highly sulfated dextran sulfate, pentosan polysulfate and heparin exerted a marked effect, while a number of intermediately sulfated GAGs such as HS, CS and DS had a moderate effect, and non-sulfated dextran and hyaloronic acid had no effect (Hasegawa et al., 1997). Interestingly, the concentration of sulfated GAGs required to stimulate tau phosphorylation was lower than that required for tau assembly and aggregation (Hasegawa et al., 1997), suggesting that tau phosphorylation may precede its assembly into NFTs. The increased phosphorylation and aggregation of tau observed in tauopathies may involve a substrate modulator effect of GAGs on tau conformation, as previously shown in other biological contexts (Faucher et al., 1988; Abdel Ghany et al., 1990).

Association of HSPGs with proteins can lead to conformational changes in the bound proteins, as demonstrated by the allosteric effect of heparin on anti-thrombin. Heparin induces a conformational change in tau (Paudel and Li, 1999; Sibille et al., 2006; Elbaum-Garfinkle and Rhoades, 2012), and heparin-induced conformational changes have been shown to expose previously masked tau phosphorylation sites (Zheng-Fischhöfer et al., 1998; Paudel and Li, 1999; Yoshida and Goedert, 2012). Electron microscopy data has provided evidence that sulfated GAGs present in tau aggregates affect paired helical filament (PHF) conformation: treatment of PHF-tau extracted from AD patients with heparinase or chondroitinase resulted in untwisting of PHF filaments (Arrasate et al., 1997). Furthermore, treatment of PHF-tau tangles with heparinase alters their immunodecoration properties, suggesting conformational changes upon heparinase treatment (Hernández et al., 2002). Specifically, heparin interacts with the second (R2) and third (R3) repeat regions of the tau microtubule-binding domain (Sibille et al., 2006). More recently, a small domain located in the N-terminal region of the R2 repeat was identified as critical for binding (Mukrasch et al., 2005; Zhao et al., 2017). Interestingly, Hasegawa et al. (1997), have suggested that sequence differences among moderately sulfated GAGs likely play a role in their interaction with tau (Hasegawa et al., 1997), as the overall level of sulfation cannot account for the observed effects. This further suggests that specific sulfated sequences on GAGs are required for interaction with tau.

Most importantly, cell surface HSs also act as receptors for Aβ (Kanekiyo et al., 2011) and extracellular tau aggregates, likely mediating a prion-like propagation of the pathology (Holmes et al., 2013), as reflected by the stages defined by Braak and Braak (1991). Membrane-associated heparan sulfate is involved in cell surface binding, uptake and internalization of tau seeds (Holmes et al., 2013) suggesting that propagation of tau seeds can also be blocked by pharmacological targeting of cell surface HSPGs. This approach may slow the progression of tauopathies (Holmes and Diamond, 2014). Alteration of heparan sulfate structure with aging may also be implicated in neurodegeneration. In particular, HS is important for adult neurogenesis by modulating FGF signaling; age-related alteration of HS binding properties in the hippocampus (Huynh et al., 2012), significantly compromises local regenerative capacities (Yamada et al., 2017). Membrane-bound HS, such as syndecans and glypicans are critical for regulation of neurogenesis (Yu et al., 2017).

Heparan sulfate has emerged as a potential multiplayer in tau pathology. HS is potentially implicated in the initiation and propagation of tau pathology and may play a role in limiting regenerative potential in dementia-susceptible regions. Further studies are needed to elucidate the role of different HSPGs in tauopathies. Memebrane-bound HS, mostly syndecan-3, syndecan-4 and glypican-3, are increased in AD brain (Liu et al., 2016). Particular attention should be paid to processes defining regional susceptibility to tauopathy.

## Zebrafish for Drug Discovery in Tauopathies

The zebrafish (*Danio rerio*), is a well-established model for the study of developmental biology, gene function and human diseases. As a vertebrate, it is closer to humans than invertebrate models, such as nematodes and fruit flies, and offers many advantages over invertebrate and other alternative vertebrate models.

The zebrafish is particularly well-suited for forward and reverse genetic and high-throughput functional studies. Major advantages of zebrafish include a precisely characterized genome with genes showing 50–80% sequence similarities with their human orthologs. Orthologous counterparts of many genes relevant to tauopathies or specifically to Alzheimer’s disease are identified in zebrafish. The zebrafish has undergone an ancient genome duplication followed by loss of many duplicated genes. Two paralogs of the human *MAPT* gene, *mapta* and *maptb*, have been identified in zebrafish. Interestingly, in early developmental stages *mapta* transcripts contain predominantly 4-6 Rs, while most *maptb* transcripts contain 3Rs (Chen et al., 2009). In embryonic stages, the expression of *mapta* and *maptb* show a positive correlation with sex-linked ubiquitin-specific peptidase 9 (USP9) (Köglsberger et al., 2017). In the adult zebrafish brain, hypoxia is reported to shift splicing of *mapta* and *maptb* transcripts toward 4-6 R isoforms (Moussavi Nik et al., 2014). Orthologs to human *APOE* (*apoea* and *apoeb*), *APP* (*appa* and *appb*) and the APP cleaving complex are also identified in zebrafish (Newman et al., 2014).

Further advantages of the zebrafish include low cost, high fecundity, short generation time, external fertilization, external embryonic development and embryo transparency allowing easy manipulation and visualization. This small fish is particularly advantageous for the study of the nervous system as its small brain size facilitates imaging. Despite differences in CNS development, anatomy and chemistry (Panula et al., 2010), overall brain organization (Rupp et al., 1996) with neuroanatomical (Rink and Wullimann, 2004; Mueller and Wullimann, 2009) and neurochemical (Mueller et al., 2004) pathways show striking similarities in zebrafish and humans. The adult zebrafish also displays high-order behaviors such as memory, social contacts and conditioned responses (Guo, 2004). In addition, the transparency and small size of its brain allow recording of neuronal activity by the recently developed molecular sensors such as genetically encoded calcium indicators or voltage sensors (Lin and Schnitzer, 2016).

Microinjection of antisense morpholino oligonucleotides (MOs) into one-cell stage zebrafish embryos allows rapid, simple and specific reverse genetic studies by inhibition of mRNA splicing or translation (Nasevicius and Ekker, 2000; Eisen and Smith, 2008). Efficient transgenesis methods and large-scale mutagenesis screens, mRNA injection, and genomic editing tools such as the CRISPR/cas9 approach are developed in zebrafish (Prykhozhij et al., 2018). Zebrafish embryos are also suitable for large-scale chemical screening to identify novel therapeutic targets and compounds. Whole cell, tissue or organism assays are twice as often successful in drug discovery efforts than target-based assays, and the ideal assay settings are whole organism assays. Phenotypic screens, particularly in CNS drug discovery, have been markedly more efficient in identifying novel therapeutic entities than target-based assays in recent pre-clinical studies (1999–2008) (Swinney and Anthony, 2011). Zebrafish embryos have also been used to study the potential toxicity of an imaging probe for NFTs (Anumala et al., 2013) and a neuroprotective agent for AD (Torres et al., 2014).

Recent successes in drug discovery in zebrafish rely on conserved physiological pathways and drug metabolism. Here we cite two examples of such discoveries. Clemizole, approved by the FDA, has been found to inhibit seizures in a zebrafish phenotypic screen for antiepileptic drugs (Baraban et al., 2013). Zebrafish larval assays have also revealed an FDA-approved substance that protects against the ototoxicity of aminoglycoside, which enters a phase I clinical trial this year (Smuga-Otto, 2018).

The analogy of brain structure and neuronal subtypes between zebrafish and humans, together with expression of key tau phosphorylating kinases, makes the zebrafish an attractive model organism for investigating tau pathology (Santana et al., 2012). Several transgenic zebrafish models of tauopathy expressing either the wild-type or mutant human tau protein are generated. These models represent the proof of concept that zebrafish models of tauopathy recapitulate key aspects of tau pathology. First, a zebrafish model transiently expressing a mutant human tau protein fused to GFP reproduced the cytoskeletal pathology in the brain including the accumulation of tau in NFT-like aggregates and the presence of hyperphosphorylated tau (Tomasiewicz et al., 2002). The hybrid protein was under the control of the pan-neuronal *gata2* promoter, which is expressed in the brain, retina and spinal cord and shows mosaic expression. This study demonstrated the conservation of pathways involved in tauopathy in zebrafish, making it possible to robustly model the pathology in this species.

More recently, a stable zebrafish transgenic line was produced that expresses the human 4-repeat tau under the control of the zebrafish *enolase-2* (*eno2*) promoter. The utility of the *eno2* promoter lies in its expression, which starts in differentiated neurons and persists into adulthood. Tau deposits resembling NFTs were found within neuronal cell bodies and proximal axons in brain regions relevant to PSP (Bai et al., 2007; Bai and Burton, 2011). Later, a stable zebrafish transgenic line expressing the human tau protein carrying the tau^P301L^ mutation linked to frontotemporal dementia (FTD) under the control of the zebrafish pan-neuronal HuC promoter was generated (Paquet et al., 2009). In this model, the Tg[HuC::hTau^P301L^/DsRed], the vector system allows expression of the mutated human tau P301L along with simultaneous expression of DsRed fluorescent reporter, facilitating the identification of the mutated tau expressing neurons. Tg[HuC::hTau^P301L^/DsRed] develops spinal motoneuron axon extention and elongation defects and neurodegeneration in early developmental stages and as a consequence deficits in the escape response reflex. Similar defects were also observed in zebrafish larvae expressing the mutant A152T tau protein (Lopez et al., 2017). The chronic expression of the P301L mutant tau into adulthood has also been recently examined in zebrafish (Cosacak et al., 2017). Focusing on the adult zebrafish brain, Cosacak et al reported tau hyperphosphorylation in the telencephalon. However, the authors did not find tauopathy-related symptoms in the telencephalon, such as formation of neurofibrillary tangles. This is in contrasts with another report (Bai and Burton, 2011) on tau mislocalization and tau-laden neurofibrillary like structures in tau transgenic adult zebrafish brain.

The observed discrepancies may be due to the specific brain regions studied, telencephalon (Cosacak et al) vs. optic tectum and brainstem (Bai and Burton), or different transgenic systems or labeling methologies used. However, the possible resistance of adult zebrafish telencephalon to tauopathy is an interesting avenue of investigation that may reveal potential protective mechanisms. Moreover, HS plays a role in nervous system regeneration (Fuller-Carter et al., 2015; Yamada et al., 2017). High regenerative capacities in zebrafish (Gemberling et al., 2013) makes the organism a suitable model to study the implication of HS in regenerative processes.

A transient expression system is also established that expresses either full-length or truncated zebrafish 3R or human 4R tau, fused to GFP, under the control of HuC promoter (Wu et al., 2016). The expression of all constructs was found to be toxic, causing significant neurodegeneration. Using this experimental system enhancing an anti-apoptotic, anti-oxidative or neurotrophic mechanisms, was found to be protective against neurodegeneration.

The zebrafish offers numerous, diverse advantages as an animal model for the study of nervous system pathology. Numerous genes orthologous to those involved in tauopathies have been identified in the zebrafish. Experiments on zebrafish have confirmed the conservation of essential pathways between humans and the zebrafish that enable it to model tau pathology. Specific brain regions in the zebrafish are reported to bear resistance to NFT formation, possibly due to high regenerative capacities. Elucidation of these mechanisms, in particular the role of HS in the pathological vs. protective or regenerative processes in zebrafish will help identify potential pathways and targets to prevent or treat tau pathology.

## Therapeutic Approaches Targeting HSPGS in Tauopathies

A better knowledge of HS-protein interactions has huge therapeutic implications for human pathologies (Capila and Linhardt, 2002; Lindahl, 2007). The primary therapeutic approach based on targeting PGs/GAGs in tauopathies consists of treatment with exogenous GAGs or GAG mimetics. Administration of exogenous GAGs is believed to competitively inhibit the harmful processes mediated by endogenous GAGs (Wang and Ding, 2014). A summary of therapeutic approaches involving exogenous GAGs and GAG mimetics is presented in Table 1. Indeed, LMW heparin oligomers efficiently inhibit the stimulatory effect of heparin on APP secretion, and inhibit the binding of heparin to Aβ_1-28_ peptide *in vitro* (Leveugle et al., 1998). However, large sulfated GAGs such as heparin and DS inhibit the binding of HSPGs to APP *in vitro* (Narindrasorasak et al., 1991). Several polysulfated GAGs and synthetic sulfate-containing compounds such as the Aβ-binding sulfonated dye Congo Red, along with several other sulfonated dyes, have been found to attenuate the neurotoxic effects of Aβ_25-35_ and Aβ_1-40_ *in vitro* (Pollack et al., 1995a,b). Moreover, several small anionic substances with sulfonate and sulfate residues efficiently decrease Aβ deposition (Kisilevsky et al., 1995). Accordingly, arrays of LMW GAGs are developed as competitive inhibitors of PG interactions (Gervais et al., 2001). Neuroparin (C3), an LMW GAG derivative of heparin, has been shown to have neuroprotective and neuroreparative properties in several animal models of AD (Dudas et al., 2008). Interestingly, neuroparin is reported to attenuate the abnormal tau immunoreactivity (representative of an AD-related conformational alteration of tau) in rat hippocampus following unilateral injection of Aβ_25-35_ into the amygdala (Dudas et al., 2002). In addition, this abnormal Aβ_25-35_ induced tau immunoreactivity was fully prevented following chronic subcutaneous injections of the LMW GAG ceroparin (Walzer et al., 2002). Heparin-like oligosaccharides reduce uptake of tau oligomers limiting their infectivity (Wang et al., 2018).

**Table 1:** Classic strategies targeting HSPGs in tauopathies.

Category		Names	Targets	Settings/Organisms	Outcomes	References
Sulfated glycosaminoglycans (GAGs)	Conventional GAGs	Heparin, dextran sulfate and other GAGs	APP/Aβ	*In vitro*, PC12 cells	Inhibition of HSPG binding to APP	Narindrasorasak et al., 1991; Leveugle et al., 1994
		Heparin analogs	BACE1	*In vitro*	BACE1 inhibition	Patey et al., 2006, 2008
	Low molecular weight (LMW) heparins	Heparin oligosaccharides	APP/Aβ	*In vitro*, Human	Inhibition of the stimulatory effect of heparin on APP secretion and heparin binding to Aβ_1-28_	Leveugle et al., 1998
			Tau	Neuroblastoma culture	Reduction in cellular uptake and cytotoxicity of tau oligomers	Wang et al., 2018
		Enoxaparin/Dalteparin	Aβ	*In vitro*	Disassembley of Aβ_40_ fibrils	Zhu et al., 2001
		Certoparin/Neuroparin (C3)	Aβ/Tau	Rat	Reduction in tau abnormal conformation in hippocampus, stimulated by Aβ_25-35_ intra-amygdaloid injection	Walzer et al., 2002/ Dudas et al., 2002, 2008
		Enoxaparin	Aβ	APP23 mice	Decrease in Aβ brain concentration, deposition and reactive astrocytosis	Bergamaschini et al., 2004
				APPswe/PS1dE9 mice	Improvement of spatial memory, decrease in Aβ deposition if treated in early stages of Aβ accumulation	Timmer et al., 2010
Low molecular weight anionic sulfonate or sulfate compounds		Congo red and analogs	Aβ	PC12/Hela cells	Attenuation of Aβ_25-35_ and Aβ_40_ toxicity	Pollack et al., 1995b,a
		Sodium 1,3-propanediol disulphate	Aβ	*In vitro*	Disassembly of Aβ_40_ fibrils	Kisilevsky et al., 1995
		Tramiprosate (3-amino-1-propanesulfonic acid; 3APS; homotaurine; Alzhemed^TM^)	Aβ	TgCRND8 mice	Significant reduction of Aβ in brain and plasma, reduction of Aβ plaques in brain	Gervais et al., 2007
				Humans: Phase II trial in probable mild to moderate AD	Satisfactory safety and tolerability, significant decrease of CSF Aβ_42_ levels, no cognitive benefits	
				Phase III trial in mild to moderate AD	No cognitive benefits, *post-hoc* analyses: decrease in loss of hippocampal volume	Aisen et al., 2006
					Cognitive benefits in APOE4/4 patients	Abushakra et al., 2017

Another ionic compound, tramiprosate (3-amino-1-propanesulfonic acid; 3APS; Alzhemed^TM^), has been shown to bind preferentially the soluble Aβ peptide, maintain Aβ peptide in a soluble conformation, and reduce Aβ burden in TgCRND8 mice, a transgenic AD model that develops early-onset and aggressive brain Aβ amyloidosis (Gervais et al., 2007). However, in November 2007, tramiprosate was rejected for further pharmaceutical development because of a lack of proven efficacy in a phase III human clinical trial (Carter et al., 2010). However, while clinical trials of tramiprosate in mild-to-moderate AD have not shown therapeutic efficacy, *post-hoc* analyses have shown some significantly positive outcomes on secondary endpoints and in some particular subgroups of patients (Aisen et al., 2011; Caltagirone et al., 2012). In particular, the disease-modifying potential is highlighted in APOE4 allele carrier patients by the observed cognitive benefits (Caltagirone et al., 2012; Abushakra et al., 2017).

**FIGURE 1:**
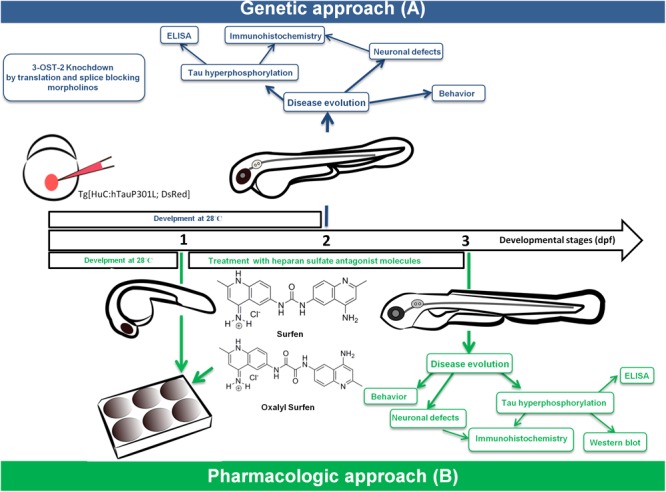
Combination of genetic **(A)** and pharmacological **(B)** approaches identified novel therapeutics for tauopathies using a zebrafish transgenic model of tau pathology.

The classic strategies aimed at HSPGs are mostly tested in AD where benefits are observed in distinct patient subgroups. In these strategies, APP and Aβ (not tau) are mostly studied as targets. An alternative approach consists in targeting HS sulfation. Recently, overexpression of several HS biosynthetic enzymes such as 3-*O*-sulfotransferase-2 (3ost2 or Hs3st2) was demonstrated in the hippocampus of AD patients. Inactivation of the zebrafish ortholog of the HS chain modifier enzyme Hs3st2 significantly decreased tau hyperphosphorylation and tau-related neuropathology *in vitro* and *in vivo* in the Tg[HuC::hTau^P301L^/DsRed] zebrafish model of tauopathy (Sepulveda Diaz et al., 2015). An additional approach consists of treatment with heparan sulfate antagonist molecules. In a recent proof of concept study a rescue of neuronal abnormalities upon treatment with surfen and oxalyl surfen was observed in the Tg[HuC::hTau^P301L^/DsRed] zebrafish model (Alavi Naini et al., 2018). Figure 1 summarizes the two approaches in Tg[HuC::hTau^P301L^/DsRed] zebrafish.

3-*O*-Sulfation in HS chains forms binding sites for HSPG binding proteins. However, biochemical characterization of this rare modification among an array of other sulfated groups requires large sample quantities, and shortage of material for studying 3-*O*-sulfation has so far hampered work on these sulfated motifs. So far six HSPG-binding proteins have been shown to require 3-*O*-sulfation for their association with GAG chains (Thacker et al., 2014).

The addition of a 3-*O*-sulfate group is one of the last chain modifications in HS biosynthesis (Zhang et al., 2001a,b). This modification is a rare and discrete modification confined to a limited number of chains when it occurs at all, and its prevalence among naturally occurring HSs is largely unknown (Thacker et al., 2014). The 3-*O*-sulfate group was initially identified while searching for sulfate group removing enzymes on heparin (Leder, 1980), and later confirmed with chemical, NMR and mass spectrometry analyses (Meyer et al., 1981; Yamada et al., 1993).

The best-studied case of 3-*O*-sulfate group-dependent binding is the association of anti-thrombin (AT) to heparin and HS. Binding of heparin to AT induces conformational changes in the protein that radically increase its catalytic activity (Rosenberg and Damus, 1973; Huntington et al., 1996). The activated AT then inactivates several proteases, including thrombin, factor IXa, and factor Xa, which are involved in blood coagulation. This is why heparin is used as a routine anticoagulant agent in clinical practice. The characterization of AT binding sites on the sulfated sugar chains is facilitated by their enrichment in heparin, which is readily available commercially (Yamada et al., 1993). The minimum AT binding site on heparin is a pentasaccharide sequence with a critical 3-*O*-sulfated motif on the middle *N*-sulfo-glucosamine residue (Thunberg et al., 1982; Choay et al., 1983). In the absence of this 3-*O*-sulfate group, the affinity of heparin to AT was markedly decreased, along with its inhibitory effect on factor Xa (Atha et al., 1985, 1987).

3-*O*-Sulfated HSs at the cell surface also serve as entry receptors for the herpes simplex envelope glycoprotein, glycoprotein D (gD) (Shukla et al., 1999), and the binding domain has been identified as an octosaccharide containing a 3-*O*-sulfated motif (Liu et al., 2002). In a similar manner, fibroblast growth factor 7 (FGF7) and fibroblast growth factor receptors (FGFR) are thought to rely on 3-*O*-sulfated motifs for binding to HS and heparin (McKeehan et al., 1999; Ye et al., 2001; Luo et al., 2006). Moreover, 3-*O*-sulfation on heparin/HS is suggested to mediate the binding of “cyclophilin B” and “stabilin” (Vanpouille et al., 2007; Pempe et al., 2012). Cyclophilin B promotes lymphocyte adhesion and migration following binding to HS located at the cell surface (Allain et al., 2002). Stabilins (1, 2) mediate heparin clearance in hepatic sinusoidal endothelial cells (Hansen et al., 2005; Harris et al., 2008).

Combination of genetic (A) (morpholino gene-KO) (Sepulveda-Diaz et al., 2015) and pharmacological (B) (small molecule) (Alavi Naini et al., 2018) approaches in the zebrafish Tg[HuC::hTau^P301L^; DsRed] tauopathy model identified 3-*OS*-sulfated HS as a therapeutic target for tauopathies and the small molecules surfen and oxalyl surfen as novel therapeutic candidates.

Alterations in the fine structure of GAGs have previously been described in several pathological situations. The sulfate moieties on GAG chains have been shown to contribute to amylin fibrillization in islet amyloidosis (Castillo et al., 1998). Furthermore, alteration of the *O*-sulfation pattern and specific disaccharide compositions has been observed in amyloid-laden liver and spleen tissues (Lindahl and Lindahl, 1997). An analysis of the HSs in the cerebral cortex in AD and control subjects demonstrated an alteration in *N*-sulfated residue distribution in AD brain, with an increase in GlcNSO3 residues (Lindahl et al., 1995). However, in studies of cerebral tissue the abnormal HSs may have been diluted with normal HSs, as analyses were performed on whole brains. In particular, alterations in specific HSPG populations or modifications of small domains in HSPG chains may have been hidden. Moreover, in AD, analysis of skin fibroblasts from patients revealed alterations in HS sulfation. Zebrower and Kieras reported that cultured skin fibroblasts from AD patients produced 30% more GlcA-GlcNSO3, and 40% less GlcA-GlcN-6-OSO3 compared to samples from age-matched healthy individuals (Zebrower et al., 1992). Authors suggested that an alteration in HS-sulfotransferase activity was responsible for their observations (Zebrower and Kieras, 1993). Importantly, a critical requirement for the presence of 6-*O*-sulfated disaccharide-containing HS in internalization and spreading of infectious tau species has recently been demonstrated (Rauch et al., 2018; Stopschinski et al., 2018). 6-*O*-Sulfation was also identified as critical for heparin-tau interaction by surface plasmon resonance (SPR) and nuclear magnetic resonance (NMR) spectroscopy (Zhao et al., 2017), while the requirement for 3-*O*-sulfation was not investigated in these recent studies. These studies together with studies on 3-*O*-sulfation (Sepulveda-Diaz et al., 2015) support the hypothesis that specific GAG chain sulfation mediated by a combination of 2-, 3- and 6-*O*-sulfotransferases play a key role in the physiopathology of tauopathies. Interestingly, hierarchical sulfation of disaccharide residues allows 3-*O*-sulfated motifs to be generated only on 6-*O*-sulfated domains of GAG chains.

Aside from hyperphosphorylation and aggregation of the tau protein, the sulfated motifs on GAG chains have also been shown to play a role in Aβ fibrillization and aggregation. This was first suggested by Fraser and coworkers, based on X-ray diffraction studies (Fraser et al., 1992). A later *in vitro* study showed that the sulfated motifs on GAG chains directly bind fibrillar Aβ (Gupta-Bansal et al., 1995). In addition, while removal of *N*-sulfated motifs on heparin GAG chains slightly reduced the heparin-induced Aβ fibril formation (Castillo et al., 1999), removal of *O*-sulfates led to a significant loss of heparin-enhanced Aβ fibrillization. Heparin enhancement of Aβ fibrillization was significantly greater than that induced by HS, consistent with the lower sulfate content in HS. By contrast, inorganic sulfate was found to have no effect (Castillo et al., 1999).

In turn, Aβ peptides have been suggested to influence GAG composition and localization (Miller et al., 1997; Timmer et al., 2009). In particular, Aβ_42_ carrying the Dutch mutation has been reported to induce cellular relocalization and production of agrin and glypican-1 *in vitro*, together with altered glycosylation and increased sulfate incorporation (Timmer et al., 2009). Interestingly, the treatment of human brain pericytes (HBPs) with Aβ_42_^Dutch^ leads to the association of Aβ fibrils to cell surface glypican-1 prior to their internalization (Timmer et al., 2009). TGF-β enhancement of Aβ fibril formation is also suggested to involve an increase in HSPG synthesis or chain modification (Castillo et al., 1999). Aβ degradation takes place in the lysosomal compartment (Li et al., 2012), the intracellular compartment where HSPG degradation also occurs (Brauker and Wang, 1987; Yanagishita and Hascall, 1992; Bame, 1993). Interestingly, Aβ has been shown to inhibit the heparanase-mediated degradation of GAGs and HSPGs *in vitro* (Bame et al., 1997), suggesting that a similar process may hamper HSPGs degradation *in vivo* in tauopathies, creating a scenario similar to that observed in patients with mucopolysaccharidoses.

Apolipoprotein E (whose E4 allele is a major risk factor in sporadic AD) promotes GAG sulfation in cultured neuroblastoma cells, and this effect is greater for ApoE4 than ApoE3 (Bonay and Avila, 2001). The interaction between HSPGs and apolipoprotein is necessary for the uptake of apolipoprotein E-containing lipoprotein by low-density lipoprotein (LDL) receptor related protein (LRP) by hepatocytes *in vitro*. Lipoprotein uptake in neurons is also mediated by the HSPG/LRP pathway (Mahley, 1996). Interestingly, apoE has been shown to interact with HSPGs through a binding site, which is structurally complementary to HS rich in *N*- and *O*-sulfate groups (Libeu et al., 2001). Moreover, the inhibitory effect of ApoE4 (not observed for ApoE3) on neurite outgrowth *in vitro* is likely mediated by the HSPG/LRP pathway (Bellosta et al., 1995). Moreover, a synthetic peptide that contain the amino acid residues 141-155, together with a 22 kDa N-terminal thrombin-cleavage fragment of apoE display significant toxicity to neurites from embryonic chick neurons *in vitro*, with apoE4 fragments exhibiting greater toxicity than apoE3. The toxic effect is likely mediated via the HSPG/LRP pathway, and treatments antagonizing HS prevent neurite degeneration (Crutcher et al., 1994; Marques et al., 1996). HSPG acts jointly with LRP1 for cell surface binding and uptake of Aβ (Kanekiyo et al., 2011). Finally, ApoE (Li et al., 2012) and LRP (Fuentealba et al., 2010) promote lysosomal trafficking of Aβ. ApoE acts in an isoform-dependent manner, with apoE3 enhancing Aβ trafficking and degradation more efficiently than apoE4 (Li et al., 2012). These observations support the hypothesis that a defect in the metabolism of HS orchestrated by Aβ and apoE may play a role in the physiopathology of tauopathies.

The internalization of HS in the intracellular space has been described in several pathological situations (Cole and Liu, 2006). The intracellular accumulation of HS long before detection of tau pathology in neurons in AD and in Down syndrome was described decades ago (Snow et al., 1990; Goedert et al., 1996). However, their involvement in the series of events leading to the abnormal tau phosphorylation and neurodegeneration has been largely disregarded, possibly because of the paradigm confining the biological roles of HSPGs to the extracellular space (Shriver et al., 2012).

The association of sulfated GAGs with tau in neurons has long been puzzling and was seen to result from leakage of newly synthesized sulfated GAGs from intracellular organelles such as Golgi apparatus, endosomes and lysosomes (Díaz-Nido et al., 2002). Interestingly, under two distinct pathological situations in neuroblastoma cultures, expression of human tau^P301L^ protein and exposure to oxidative stress, HS was localized in intracellular space. An intense intracellular uptake of membrane-associated HS and the concomitant hyperphosphorylation of the tau protein were observed (Sepulveda Diaz et al., 2015). These findings point to a possible altered metabolism and distribution of sulfated HS in tau-related neuropathologies. Regarding the molecular mechanisms linking 3-*O*-sulfated HSs and tau abnormal phosphorylation, *in vitro* data suggest that the sulfated GAGs bind to tau and not to the GSK3β tau-kinase (Sepulveda Diaz et al., 2015). Accordingly, in the presence of heparin (a highly sulfated HS), tau rapidly undergoes conformational changes that allow kinase access to epitopes otherwise inaccessible for phosphorylation (Hasegawa et al., 1997; Zheng-Fischhöfer et al., 1998; Paudel and Li, 1999; Sibille et al., 2006; Yoshida and Goedert, 2012). The substrate modulator effect of 3-*O*-sulfated HS motifs further supports the hypothesis that these sulfated polysaccharides act as molecular chaperones able to induce key conformational changes on tau protein and triggering its phosphorylation at newly unmasked tau residues. The hypothesis is further supported by the observation of an increase in hippocampal 3-*O*-sulfation with aging (Huynh et al., 2012). Recently, a GAG sulfotransferase inhibitor has been identified (Cheung et al., 2017) that sets a basic scaffold for the future development of highly specific and potent sulfotransferase inhibitors, which are promising candidates as potential therapeutics for tauopathies.

## Conclusion

Heparan sulfate-protein interactions are emerging as potential therapeutic targets in tauopathies. Previous work suggest a multifactorial role played by GAGs in the pathogenesis of tauopathies. Increased biosynthesis, internalization and/or decreased degradation of HS GAGs, or alterations in HS chain modifications likely contribute to tauopathies. These observations raise the question of whether specific motifs on HS chains are involved in binding to tau and induction of misfolded states. The zebrafish has recently emerged as an attractive animal model for Alzheimer’s disease research and provides an ideal tool for drug screening prior to clinical testing in mammals. Thanks to this small fish, HS is targeted in tau pathology by two different approaches. A critical requirement for 3-*O*-sulfated HS in tau pathology was discovered and HS antagonists (surfen and oxalyl surfen) were identified as efficient therapeutic candidates. 6-*O*-sulfated HS motifs have recently been identified as critical for tau seed internalization. 3-*O*-sulfation, a fine chain modification, is only generated in 6-*O*-sulfated regions. Together, these recent findings strengthen the hypothesis of distinct HS sequences implicated in tau misfolding, hyperphosphorylation, aggregation and propagation. These findings are steps toward defining of specific HS targets in tauopathies.

## Author Contributions

Both authors contributed to writing the manuscript.

## Conflict of Interest Statement

The authors declare that the research was conducted in the absence of any commercial or financial relationships that could be construed as a potential conflict of interest.
